# Microplankton dynamics of a coastal lagoon, Chilika: interactive effect of environmental parameters on microplankton groups

**DOI:** 10.1007/s10661-018-7049-9

**Published:** 2018-10-30

**Authors:** Manasi Mukherjee, Vettath Raghavan Suresh, Ranjan Kumar Manna

**Affiliations:** 1 Central Inland Fisheries Research Institute, Kolkata, West Bengal 700 120 India; 2 Wrocław University of Environmental and Life Sciences, ul. C. K. Norwida 25, 50-375 Wrocław, Poland; 3 Central Inland Fisheries Research Institute, Kolkata, West Bengal 700 120 India

**Keywords:** Plankton dynamics, Spatio-temporal variation, Biotic-abiotic relation, Diatoms, Dinophyceae, Tintinninae

## Abstract

Microplankton population of Asia’s largest coastal lagoon Chilika was studied for five major groups, bacillariophyceae, cyanophyceae, chlorophyceae, dinophyceae, rotifera, and tintinninae. The study reported presence of 233 species of microplankton whose average annual abundance was 1631 cells/l. The physicochemical parameters contributing to the spatio-temporal fluctuations in microplankton diversity, abundance, and community structure were identified as salinity, pH, DO, nitrate, and silicate. Salinity, transparency, depth, and silicate most explained the abundance of bacillariophyceae; nitrate, pH, and DO influenced cyanophyceae; salinity, transparency, and chlorophyll concentration influenced chlorophyceae; salinity, depth, and water temperature influenced dinophyceae; salinity, free CO2, and nitrate-influenced rotifers, whereas salinity, pH, DO, and depth influenced tintinnids. Biotic-abiotic relationships revealed particular preference of environmental conditions at species level in groups like bacillariophyceae, cyanophyceae, and dinophyceae. Although the lagoon is shallow, bacillariophyceae-environment interaction showed depth can be a critical factor for species like *Aulocoseira* sp., *Amphipleura* sp., and *Rhophalodia* sp. Species of dinoflagellates like *Dinophysis caudata*, *Noctiluca scintillans*, and *Protoperidinium* proliferated in lower level of silicate. Unlike other cyanophyceae species *Streptococcus* sp., *Chroococcus* sp., *Diplococcus* sp., *Aphanocapsa* sp., and *Gloeocapsa* sp. were negatively influenced by nitrate concentration. The study provides better scope for ecological management of the lagoon with respect to conserving biodiversity and hydrological quality of the ecosystem.

## Introduction

Apart from assessing the species diversity, biologists have often deliberated on solving the “how,” “why,” and “what” questions on changes in abundance, distribution, and partitioning of ecosystems by plankton. Several abiotic and biotic factors influence plankton diversity, community structure, and spatio-temporal variations (Battish 1992). Studying environmental parameters and their influence on community structure of microplankton can help in dealing appropriately with undesirable changes in the environment. Studying the relationship between plankton community (representing structure) and plankton production (representing function) is essential to understand any ecosystem (Duarte et al. 2006). Ecosystems with marked biodiversity changes at seasonal time scales are easier to relate with ecosystem functioning (Queiroga et al. 2007). Studies have suggested that increases in diversity are associated with decreases in biomass and production (Krebs 1994). Plankton assemblage is a reflection of intra and inter-specific interactions with abiotic components (Hughes 2000) like light, temperature, inorganic and organic nutrients and biotic factors like competition and predation, which are important in regulating growth and succession of plankton communities in aquatic ecosystem (Goldman and dan Horne 1983; Wetzel 2000). Each of these criteria exerts individual and synergistic functional properties with the biological environment. For instance, phosphorus and nitrogen have been considered as the most important inorganic nutrients for growth of plankton (Sterner et al. 1995). Increase in silica leads to replacement of green algae with diatoms and *vise versa* (Likens 2010) and pH limits growth of many oligotrophic algae (Goldman and dan Horne 1983). But these environmental factors do not function independently, and have complex interdependency and affect plankton growth. For example, pH specificity in growth of plankton species varied between oligotrophic and eutrophic lagoons (Moss 1972).Variation in silicate and phosphate ratio are known to determine diatom and cyanobacterial growth (Holm and Armstrong 1981). Thus not only monitoring the changes in nutrient concentration seems important to abundance, growth, periodicity and distribution of microplankton, the related environmental variables also become equally important. Dynamic aquatic systems like coastal lagoons need continuous monitoring of these environmental variables to understand their effect on biotic components. The present study was conducted in Chilika, a coastal lagoon that constantly receives input from two varied types of aquatic systems (freshwater from rivers and saline water from Bay of Bengal).This continuously changing environment is important in determining periodic dynamics of the water parameters and influences distribution and abundance cycle of planktonic life in the lagoon. Known for its high biodiversity (Devasundararn and Roy 1954; Mohanty and Adhikary 2013; Mukherjee et al. 2015, 2016) and dynamic changes (Srichandan et al. 2015), this Ramsar site that has been recently restored from its depleting condition (Mohanty and Adhikary 2013) and thus needs constant monitoring of biotic-abiotic interactions. Though often suggested, sparse knowledge on extent of environmental influence on plankton groups and dynamics are available (Srichandan et al. 2015). To comprehend the differences that exist, and what drives the community structure of microplankotn in the lagoon, there is a need to study the causes for such changes and dynamics. Thus changes and influence of environmental parameters on microplankton populations of the lagoon were studied. The aim of this study was to identify environmental parameters governing spatial and temporal differences in microplankton abundance and composition in Chilika lagoon and to understand how specific microplankton groups can enable better identification of most crucial environmental parameters in such ecosystems.

The present study deals with the spatio-temporal changes of microplankton populations at species level and emphasize on how the species interactions can participate in determining the dynamics of the lagoon. Ecosystem management based these microplankton dynamics and biotic-abiotic interactions have been discussed in details.

## Materials and methods

### Sample collection and analysis

**Fig. 1:**
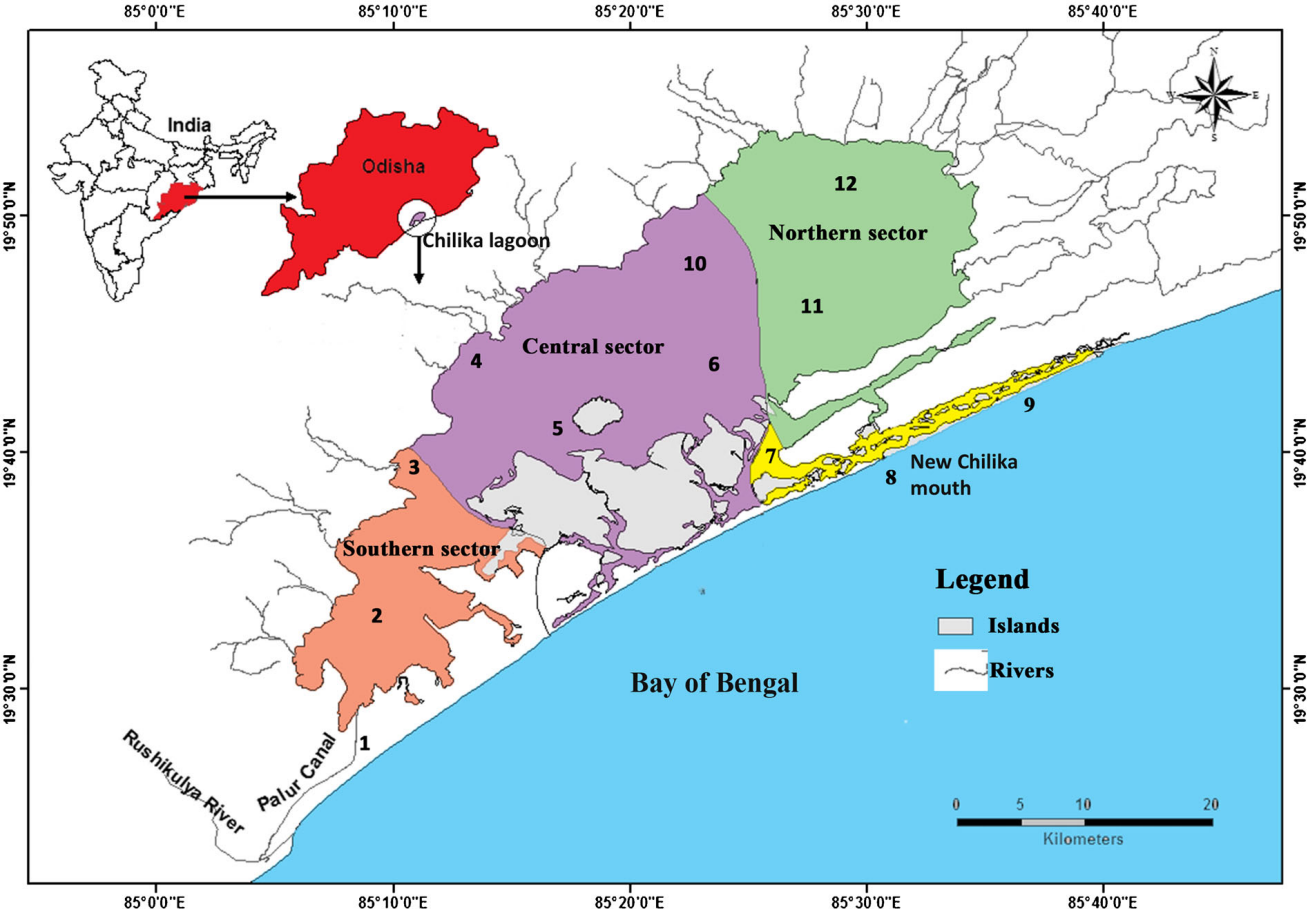
Chilika map with studied sectors stations denoted

The Chilika, is Asia’s largest lagoon located in Odisha, Inida (19′ 28° to 19′ 54° N and 85′ 50 to 85′ 38° E). Being located in coastal region, it receives saltwater through the sea-mouth and freshwater through a number of rivers. Based on the depth and salinity profile, the lagoon has been demarcated into four major sectors (Ghosh and Pattnaik 2005), namely the northern sector (freshwater), southern (saline zone), central sector (brackish zone), and outer channel sector (saline zone) (Fig. 1) and the seasons were classified into premonsoon (March–June), monsoon (July–October), and postmonsoon (November–February) for temporal study. Plankton and water samples were collected concurrently from these four sectors covering 12 stations as detailed in Fig. 1, during October 2012 to May 2015. The samples were collected following EPA (2002) and analyzed following standard methodologies of Eaton et al. (2005).

Plankton samples were collected using plankton net made of no. 19 grade cloth (74 μm mesh) with 0.5-m diameter mouth, fitted with a flow meter (General Oceanics mechanical flowmeter, model no. 2030R). The net was hauled horizontally for distance of 10 m ensuring captures of larger plankton. To compensate the loss of plankton diversity below 74-μ size, a 20-μ mesh plankton net was used by filtering 100 l of water from the same sampling points. The samples were fixed, labeled, and brought to the laboratory for analyzing their diversity and abundance. Both the samples were distinguished and treated separately. Samples of 20 to 200 μm (microplankton) size range were examined and enumerated using a Nicon Eclipse 50i microscope having image processing features. Taxonomic identification up to genus or species level was based on published keys for bacillariophyceae (Cremer et al. 2007), for cynaophyceae (Rai and Misra 2010), for dinoflagellates (Faust and Gulledge 2002; Taylor 1976; Tomas and Hasle 1997), for rotifers (Shiel 1995), and for tintinnids (Daday 1887; Hada 1932; Jorgensen 1927; Kofoid and Campbell 1929; Meunier 1919). For quantitative estimation, Sedgwick rafter cell method was followed and the abundance was quantified as (*N* = *n* × *v*/*V*), where *N* = total number of plankton cells per liter, *n* = average no. of plankton cell in 1 ml of sample, *v* = volume of plankton concentrate, and *V* = total volume of water filtered.

Water parameters were analyzed following Eaton et al. (2005). Water temperature was measured using Celsius thermometer, depth using electronic depth meter of 0.5 m accuracy, pH using a digital pH meter, transparency using a Secchi disc, salinity, and specific conductivity with EC meter (Multi Line P4 Universal meter, WTW 82363 model); dissolved oxygen following Winkler’s method and free CO_2_ using phenolphthalein indicator followed by N/44 sodium hydroxide titration. Biochemical oxygen demand (BOD) was measured after 5 days incubation in 20 °C. Water samples for nutrient parameters (silicate, nitrate, and phosphate) were collected in clean amber-colored HDPE bottles and transported to the laboratory on ice. Samples were processed through standard procedures and the absorbance was measured at 410-, 880-, and 410-nm wavelength for available NO_3_, PO_4_, and SiO_4_ respectively on UV-visible spectrophotometer.

### Data analysis

To assess the spatio-temporal variations in abundance present within the species of each group, their population throughout the period of study were segregated into the four ecological sectors of the lagoon season wise. These were then plotted to find variations, if any, both spatially and seasonally. Principal component analysis (PCA) is performed using “R” software, with the average plankton abundance and the environmental parameters of the corresponding period to address the causes and reasons of spatio-temporal patterns. The values were pretreated with log transformation to minimize the effect of variations of different units of measurement in case of environmental parameters. With the most influential environmental parameter determined from PCA, the parameters most explaining the abundance of major groups of microphytoplankton and microzooplankton species were determined through multivariate statistical approach using canonical correspondence analysis (CCA), using CANOCO software. The data prior to analysis was log transformed for normalization as various units of environmental and biological parameters were used. With 999 simulations at 5% significance and randomized residuals, the maximum explained variations were finalized.

## Results and discussion

**Fig. 2:**
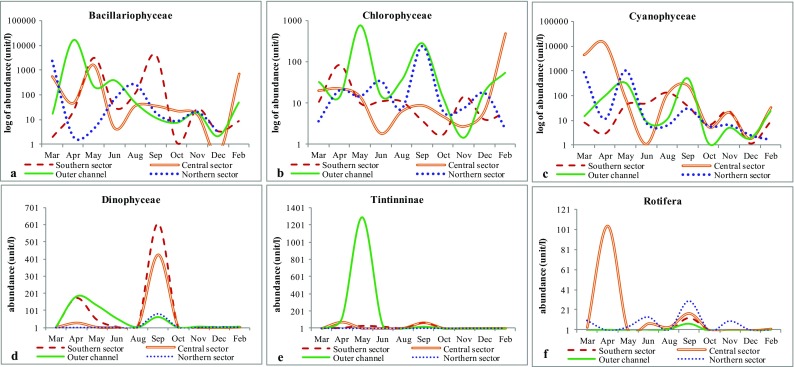
Monthly variation in group wise abundance of microplankton in the lagoon **a** bacillariopyaceae, **b** chlorophyceae, **c** cyanophyceae, **d** dinophyceae, **e** tintinninae, and **f** rotifera

The study recorded 233 species of microplankton comprising 171 species of microphytoplankton (bacillariophyceae, cyanophyceae, chlorophyceae, and dinophyceae) and 62 species of microzooplankton (tintinnina and rotifera). Among total plankton, microplankton fraction formed 73% (annual average 1631 ± 6935cells/l), wherein microphytoplankton was 1551 ± 6886 cells/l and microzooplankton was 80 ± 352 cells/l. Each microplankton group (Fig. 2) and corresponding water parameters (Table 1)recorded distinct spatio-temporal variations As reported in total plankton abundance by Adhikary and Sahu (1992), the overall microplankton abundance declined from premonsoon period to postmonsoon (Fig. 2). The outer channel sector recorded highest salinity (32.4 ppt) and depth (4.1 m), whereas northern sector had lowest depth (1.5 m) and was completely freshwater (0.00 ppt). Water temperature, salinity, and specific conductivity showed considerable rise during April to June (premonsoon), whereas the nutrient contents increased during monsoon with the onset of rainfall and gradually declined through postmonsoon to premonsoon. Of all the parameters analyzed, pH, depth, and DO showed least seasonal or temporal fluctuations. The corresponding spatio-temporal variations among each microplankton groups are discussed as follows.

**Table 1:** Seasonal and spatial variation in water parameters of the lagoon during study period

		Mar.	Apr.	May.	Jun.	Aug.	Sep.	Oct.	Nov.	Dec.	Feb.
Water temp (°C)	SS	28.5	30.0	34.0	33.3	32.6	31.1	30.9	28.7	27.2	26.3
	CS	28.5	29.8	32.7	33.2	32.5	31.4	32.1	27.7	26.3	25.7
	OC	28.2	28.6	31.8	32.6	31.7	31.2	30.6	26.8	25.0	25.6
	NS	28.0	29.4	31.3	32.3	32.2	32.3	32.5	25.9	24.1	26.2
Depth (m)	SS	1.6	2.0	2.1	1.9	2.9	2.6	1.8	2.2	1.8	1.9
	CS	1.1	1.2	1.7	1.7	2.5	2.1	1.9	1.8	1.8	1.6
	OC	2.4	2.2	1.9	2.7	3.3	2.8	2.4	2.4	4.1	2.7
	NS	1.4	1.1	1.5	1.2	1.8	1.7	1.4	2.1	1.3	2.0
Transparency (cm)	SS	53.7	69.0	89.7	45.0	85.0	93.3	45.0	72.3	86.3	98.3
	CS	45.8	99.5	76.3	40.3	60.0	69.0	42.8	67.8	87.8	109.5
	OC	53.3	71.0	80.0	44.7	23.7	52.3	42.0	61.0	67.3	96.7
	NS	35.0	44.0	54.5	26.0	20.0	64.5	24.5	24.5	64.5	72.5
Sp. cond. (mS/cm)	SS	13.3	22.9	35.1	19.7	20.7	16.7	17.7	13.3	18.1	15.2
	CS	9.2	16.6	42.8	33.2	5.1	5.3	7.8	6.2	8.7	9.2
	OC	15.2	36.6	49.2	45.3	1.5	3.2	6.5	6.5	17.9	29.2
	NS	7.5	13.6	26.0	20.6	0.2	0.3	0.4	0.9	0.6	6.0
PH	SS	8.7	8.8	8.0	8.5	8.3	8.0	7.7	8.6	9.0	8.9
	CS	9.3	9.3	8.3	8.5	8.2	8.2	8.6	8.7	8.9	9.7
	OC	8.5	7.9	8.3	8.4	8.0	8.0	8.1	8.4	8.7	8.6
	NS	8.9	7.9	8.3	8.5	8.0	8.2	8.8	8.5	7.7	8.9
DO (ppm)	SS	7.7	6.9	5.3	6.5	6.5	6.5	6.6	7.3	7.1	7.6
	CS	9.1	8.0	6.0	6.2	6.2	7.4	6.4	7.0	8.6	8.6
	OC	7.0	6.6	6.0	5.9	5.9	6.3	6.8	7.5	7.5	7.3
	NS	7.0	6.7	6.2	6.3	5.8	7.0	6.7	7.4	5.0	7.8
BOD (ppm)	SS	1.5	1.3	0.8	0.7	0.3	2.0	0.8	0.3	0.7	0.4
	CS	1.6	1.1	0.6	0.5	0.5	1.9	0.8	0.4	0.8	0.7
	OC	1.3	1.2	0.8	0.5	0.5	1.6	0.6	0.4	1.1	0.9
	NS	0.5	0.5	0.8	0.6	0.5	1.8	0.7	0.5	0.6	0.6
Bi-carbonate Alk (ppm)	SS	NA	98.0	106.3	91.3	74.0	86.3	108.0	72.0	85.3	86.7
	CS	NA	63.5	84.0	83.5	67.8	50.5	84.0	64.5	75.5	62.0
	OC	NA	104.0	80.0	75.7	64.7	55.7	81.3	79.0	89.3	61.3
	NS	NA	79.0	86.0	68.5	63.0	54.5	67.5	69.0	85.0	80.0
Total Alkalinity (ppm)	SS	66.7	128.7	122.3	120.7	96.7	103.0	121.3	108.0	104.0	146.7
	CS	56.0	110.5	120.0	104.5	74.8	77.5	94.0	92.5	104.5	121.0
	OC	58.0	124.0	114.7	107.7	64.7	74.0	86.7	89.0	98.7	116.0
	NS	60.0	105.0	110.0	92.5	63.0	68.5	73.5	81.0	86.0	100.0
Free CO_2_ (ppm)	SS	0.0	12.0	2.3	0.0	0.0	0.0	0.0	0.0	0.0	0.0
	CS	0.5	0.0	0.0	0.0	3.2	0.0	8.0	1.0	0.0	0.0
	OC	0.7	10.0	0.0	0.0	5.0	0.0	2.0	2.5	8.0	0.0
	NS	0.0	0.0	0.0	0.0	4.3	0.0	2.0	0.0	20.0	2.0
Total hardness (ppm)	SS	2266.7	4200.0	6400.0	3333.3	3733.3	2800.0	2150.0	3533.3	2733.3	2433.3
	CS	1787.5	3013.8	8075.0	5500.0	789.0	875.0	1175.0	1419.0	1150.0	1625.0
	OC	2866.7	8733.3	9066.7	6866.7	171.3	384.7	800.0	1305.0	2216.7	4333.3
	NS	1146.0	2750.0	6350.0	3592.0	59.0	70.0	261.0	126.0	102.0	1080.0
Salinity by sp. cond. (ppt)	SS	7.8	13.9	24.8	11.9	12.5	10.0	10.7	7.8	10.7	9.9
	CS	5.3	9.9	27.8	21.0	2.8	2.9	4.5	3.4	4.9	5.2
	OC	8.8	27.9	32.5	29.6	0.6	1.6	3.6	3.6	10.8	18.2
	NS	8.5	8.2	17.0	13.1	0.0	0.0	0.0	0.2	0.1	3.3
Phosphate—P (ppm)	SS	0.0	0.0	0.0	0.0	0.0	0.0	0.1	0.1	0.0	0.0
	CS	0.0	0.0	0.0	0.0	0.1	0.1	0.0	0.0	0.0	0.0
	OC	0.0	0.0	0.0	0.0	0.0	0.1	0.0	0.0	0.0	0.0
	NS	0.1	0.0	0.0	0.0	0.1	0.2	0.1	0.0	0.1	0.1
Nitrate—N (ppm)	SS	0.3	0.2	0.0	0.5	0.5	0.5	0.1	0.6	0.2	0.8
	CS	0.2	0.1	0.0	0.4	0.6	0.4	0.0	0.4	0.4	0.3
	OC	0.2	0.2	0.0	0.6	0.5	0.5	0.1	0.5	1.5	0.1
	NS	0.1	0.1	0.0	0.4	0.8	0.4	0.0	0.4	2.8	0.2
Silicate—Si (ppm)	SS	8.5	3.8	2.6	6.9	6.5	5.6	5.7	8.9	2.6	4.8
	CS	7.4	2.8	2.9	4.3	11.8	13.8	8.2	10.3	4.4	4.9
	OC	8.0	1.6	3.9	3.6	16.7	12.6	7.5	10.0	4.7	3.8
	NS	11.1	5.7	4.4	7.2	20.8	23.3	13.4	16.6	7.6	4.4
Chlorophyll	SS	3.8	78.4	21.1	54.7	4.7	7.0	0.2	11.1	0.9	2.1
	CS	5.6	84.9	21.8	29.6	10.9	13.2	0.3	4.8	3.7	4.4
	OC	6.8	47.1	18.9	26.8	36.3	7.2	0.3	7.9	4.8	3.6
	NS	5.8	45.1	25.0	37.6	14.8	12.6	0.3	22.6	10.5	9.0

*NA = Not available

### Bacillariophyceae

**Fig. 3:**
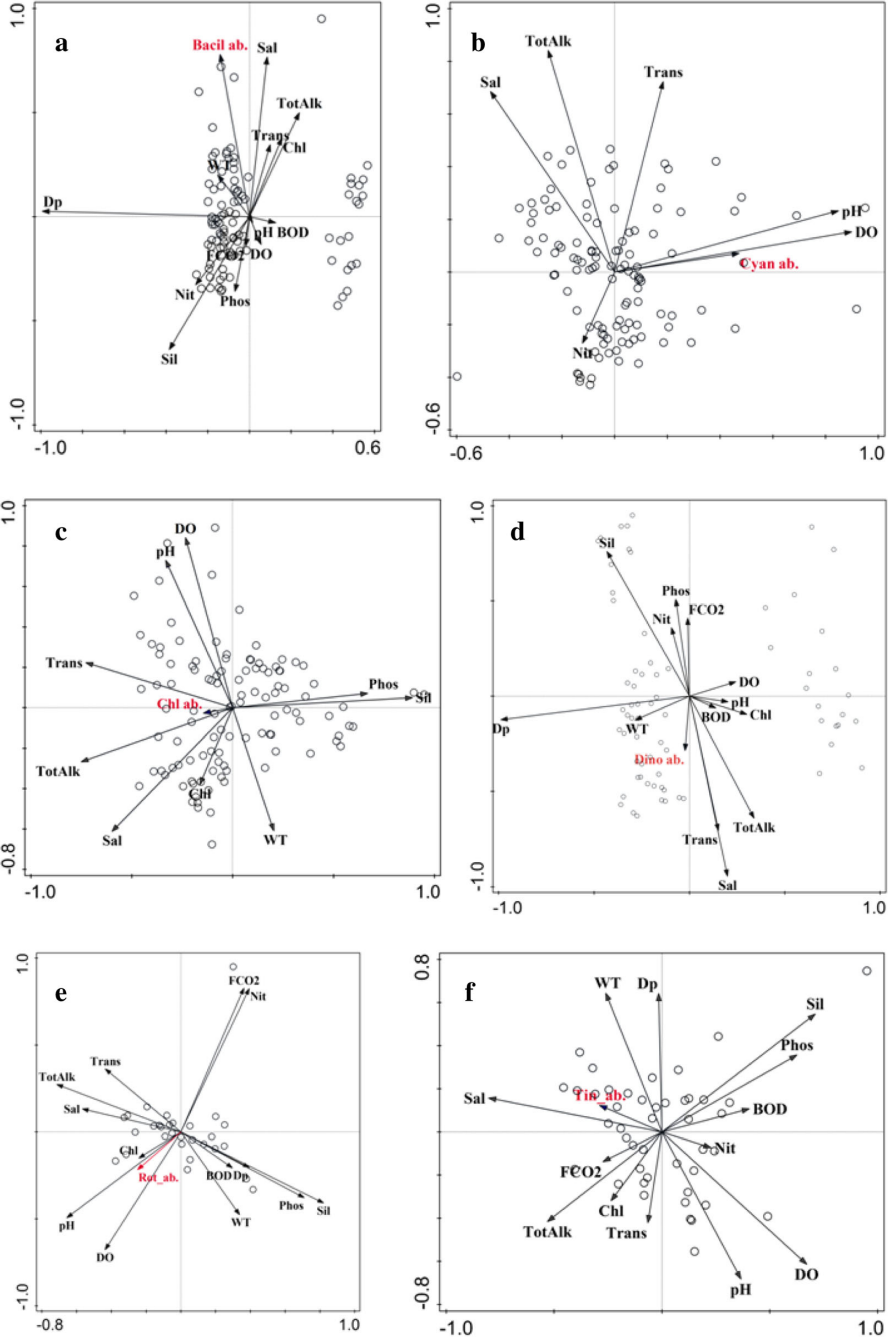
Ordination diagram showing influence of environmental parameter on microplankton abundance with principal component axis 1 (PC1) and principal component axis 2 (PC2). Bacillariophyceae (**a**), cyanophyceae (**b**), chlorophyceae (**c**), dinophyceae (**d**), rotifera (**e**), tintinninae (**f**)

**Fig. 4:**
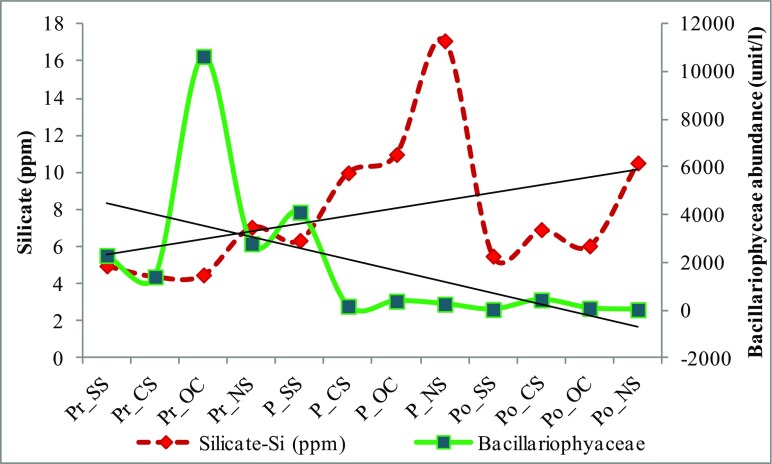
Seasonal and sector wise changes in bacillariophytes abundance and silicate concentration

**Fig. 5:**
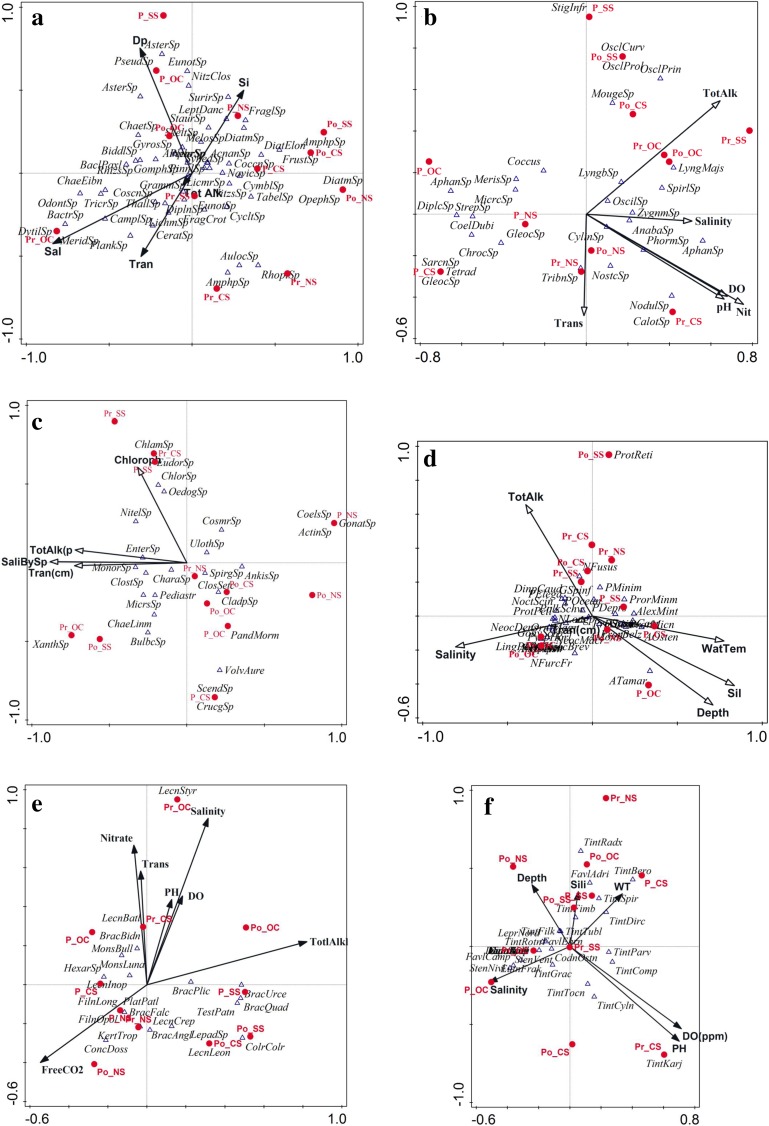
Triplot ordination of bacillariophyceae (**a**), cyanophyceae (**b**) chlorophyceae (**c**), dinophyceae (**d**), rotifera (**e**), and tintinninae (**f**) based on CCA with CC1 and CC2. The environmental variables are indicated by arrows in bold, samples in red with classified seasonal stations and species are denoted in italics. Pr = premonsoon, P = monsoon, Po = postmonsoon, NS = northern sector, CS = central sector, SS = southern sector, OC = outer channel

In congruence with other studies of the lagoon (Adhikary and Sahu 1992; Mohanty and Adhikary 2013; Rath and Adhikary 2005; Srichandan et al. 2015), bacillariophyceae was the most dominant microphytoplankton group followed by cyanophyceae and chlorophyceae in the lagoon (Fig. 2a–c). Though, outer channel showed the highest dominance of this group during premonsoon, from Fig. 2a, it is evident that the group varies more temporally than spatially. The effect of seasonality studied through PCA showed that, the first two principal components could explain 62% of the variation (Table 2). Bacillariophyceae abundance showed strongest relation with salinity and depth (Fig. 3, PC2 of Table 3). Bacillariophyceae, being photosynthetic or autotrophic, are restricted to photic zones and thus their abundance is known to decrease with depth (Cantonati et al. 2009). But, Chilika is shallow lagoon (average depth of 1.01 to 2.27 m) and thus outer channel although is the deepest sector of the lagoon (Table 1) was most abundant in bacillariophyceae (Fig. 2) corresponding to a strong influence of depth on bacillariophyte abundance (Fig. 3a and Table 3). Bacillariophytes also showed a strong negative relation with silicate (Table 3 and Fig. 3a). The silicate concentration, when observed during the study, showed a completely opposite trend to the bacillariophyaceae abundance in the lagoon (Table 1). As also reported by Adhikary and Sahu (1992), highest concentration of silicate and lowest abundance of bacillariophyceae (Fig. 4) was recorded from northern sector followed by central sector, outer channel, and southern sector. Indeed, the sector (Southern sector) with least concentration of silicate had highest abundance of bacillariophyaceae (Fig. 4) during monsoon. This reciprocal relation can be a response of bacillariophyaceae utilizing the silicate for construction of their thecate cell wall or frustules (Horner 2002). Thus, the bacillariophytes flourish utilizing silica, increasing their abundance, and decreasing the silicate concentration in the environment, indicating that nutrients regulate the spatial phytoplankton distribution in an ecosystem, as also described by Egge and Aksnes (1992). Species specific effect of these environmental parameters was examined through the ordination diagram (Fig. 5a), which showed two major clusters of bacillariophyceae; one with depth and the other with salinity, transparency, and silicate. *Asterionellopsis* sp., *Eunotia* sp., *Nitzschia closterium*, *Pseudonitzschia* sp. and *Asterionella* sp. were more influenced by and dependent on depth of the lagoon. Similarly distribution of *Dytilum* sp.,*Chaetoceros* sp., *Odontella* sp., *Planktionella* sp., *Bacteriastrum* sp. were positively related to salinity. Though *Fragilaria crotonensis*, *Diploneis* sp., *Licmorpha* sp., etc. showed affinity to high salinity, their distribution was more influenced by transparency than other parameters in the lagoon. *Aulocoseira* sp., *Amphipleura* sp., and *Rhophalodia* sp. formed another cluster, abundant in northern and central sector during premonsoon. *Surirella* sp., *Fragilaria* sp., *Diatoma* sp., *Melosira* sp., *Acnanthes* sp., *Cocconeis* sp. etc. were more related to silicate concentration than other parameters.

**Table 2:** PCA results of environmental variables versus abundance of microplankton groups

Plankton groups	PCA results	Axis 1	Axis 2	Axis 3	Axis 4
Bacillariophyaceae	Eigenvalues	0.375	0.244	0.144	0.139
	Explained variations (cumulative)	37.57	61.99	76.42	90.33
Cyanophyaceae	Eigenvalues	0.29	0.265	0.153	0.125
	Explained variations (cumulative)	29.79	56.34	71.67	84.18
Chlorophyceae	Eigenvalues	0.29	0.23	0.12	0.09
	Explained variations (cumulative)	29.40	51.94	63.85	73.38
Dinophyceae	Eigenvalues	0.46	0.21	0.16	0.07
	Explained variations (cumulative)	45.76	66.41	82.23	89.84
Rotifera	Eigenvalues	0.33	0.26	0.14	0.08
	Explained variations (cumulative)	32.56	58.60	72.18	80.28
Tintinninae	Eigenvalues	0.21	0.19	0.13	0.08
	Explained variations (cumulative)	20.79	39.52	52.26	60.61

**Table 3:** Loadings or correlation coefficients of PCA

	Bacillariophyceae		Cyanophyceae		Chlorophyceae		Dinophyceae		Rotifera		Tintinninae	
	PC1	PC2	PC1	PC2	PC1	PC2	PC1	PC2	PC1	PC2	PC1	PC2
Water temperature (°C)	− 0.002	0.000	− 0.128	− 0.328	− 0.110	− 0.362	− 0.158	− 0.427	0.178	0.276	− 0.180	− 0.385
Depth (m)	− 0.387	0.830	− 0.160	− 0.286	− 0.134	− 0.161	− 0.208	− 0.205	0.208	0.118		− 0.389
Transparency (cm)	0.087	0.060	0.415		0.391		0.408		− 0.230	− 0.210		0.266
pH	− 0.002	− 0.006	0.213	0.337	0.238	0.457	0.224	0.463	− 0.347	0.286	0.240	0.408
BOD (ppm)	0.012	− 0.150		0.258		0.152			0.157	0.120	0.234	
DO (ppm)	− 0.010	− 0.018		0.422	0.187	0.551	0.174	0.510	− 0.231	0.393	0.417	0.352
TotAlk (ppm)	0.053	− 0.026	0.398		0.418	− 0.207	0.406	− 0.199	− 0.377	− 0.159	− 0.297	0.280
FCO2 (ppm)	0.090	0.066	0.292		− 0.167	− 0.129	− 0.124		0.192	− 0.479		
Salinity (ppm)	0.292	0.039	0.426		0.319	− 0.416	0.302	− 0.383	− 0.300		− 0.477	
Phosphate (ppm)	− 0.110	− 0.126	− 0.133	0.432	− 0.362	0.113	− 0.363	0.136	0.374	0.219	0.364	− 0.253
Nitrate (ppm)	− 0.238	0.219	− 0.165		− 0.143		− 0.126	0.185	0.208	− 0.478	0.131	
Silicate (ppm)	− 0.240	0.035	− 0.458		− 0.504		− 0.504		0.434	0.237	0.401	− 0.370
Chlorophyll (ppm)	0.231	0.259	0.154	− 0.258		− 0.215		− 0.166	− 0.128		− 0.119	0.202

### Cyanophyceae

Spatially, cyanophytes were found most abundant in central sector, while seasonally during premonsoon period (Fig. 2c). Known from many aquatic systems (Branco et al. 2001), DO, pH, salinity, total alkalinity, and transparency were more influencing in determining cyanophycaea abundance (Fig. 3b). Though not significant, a negative relation is indicated between the nitrate and cyanophyte population in the lagoon (Table 3), while a very significant positive relation was recorded with transparency (Fig. 3b and Table 3), especially determining the species distribution in northern sector (Fig. 5b).Two different clusters were formed of the CCA analysis indicated temporal distinctness; one with monsoon and another with premonsoon and postmonsoon (Fig. 5b). Freshwater species, *Aphanocapsa* sp., *Merismopedia* sp., *Microspora* sp., *Coelospaerium* sp., found during monsoon period, were negatively related to salinity mostly. Distribution of *Stigonema informe*, *Oscillatoria princeps*, *Oscillatoria curviceps*, and *Mougeotia* sp. were most closely related to total alkalinity. *Anabaena* sp., *Nostoc* sp., *Aphanocapsa* sp., *Phormidium* sp., *Nodularia* sp. were more dependent on nitrate content, alkalinity, and DO of the lagoon. Nitrate, as an important nutrient parameter for cyanophytes to flourish have also often been emphasized (Olden 2000) that are known for nitrifying property. Nutrient parameters of the lagoon, especially nitrate concentration influenced (Fig. 3b) spatio-temporal changes of cyanophyte abundance of the lagoon. All coccus groups (*Streptococcus* sp., *Chroococcus* sp., *Diplococcus*sp., *Aphanocapsa* sp., *Gloeocapsa* sp., etc.) of cyanophytes known for nitrate reducing property (Wyatt and Silvey 1969) formed an assemblage that exhibited significant negative correlation with nitrate.

### Chlorophyceae

The chlorophyceae were found to be more abundant in outer channel followed by northern sector and showed lesser temporal variation as compared to bacillariophceae and cyanophyceae (Fig. 2). Chlorophyceae abundance did not show significant relation with the environmental parameters, as only 52% of the variations were explained (Table 2) in the PCA analysis. But the PCA loadings showed that, it was negatively influenced by nutrient parameters like phosphate and silicate, whereas positively related with pH and DO. Considering microplankton do not account for the total chlorophyll filtered through 0.45-μm filter, a positive relation of chlorophytes with chlorophyll was recorded but was not significant (PC 2 loadings in Table 3).With only 61% variation explained (Table 4), Chlorophyceae showed three major clusters from CCA (Fig. 5c); one with chlorophyll, second (*Monoraphidium* sp., *Pediastrum* sp., *Closterium* sp., *Enteromorpha* sp., and *Microspora* sp.) positively and third (*Spirogyra* sp., *Closterium* sp., *Cladophora* sp. and *Ankistrodesmus* sp.) negatively related to salinity and transparency and during premonsoon and postmonsoon period occurred in clear water. On the contrary *Spirogyra* sp., *Closterium* sp., *Cladophora* sp. and *Ankistrodesmus* sp. need higher dissolved solids for growth (Eiseltová 2011), thus tend to spread the filaments to cover the water surface; resulting in negative relation. They also showed negative relation to salinity indicating their lesser tolerance to salinity and were more abundant in freshwater areas.

**Table 4:** CCA statistical results (eigenvalues) of microplankton groups

Plankton	Bacillariophyaceae	Cyanophyaceae	Chlorophyceae	Dinophyceae	Rotifera	Tintinninae
Axis 1	0.32	0.51	0.34	0.49	0.76	0.46
Axis 2	0.21	0.22	0.27	0.26	0.68	0.39

### Dinophyceae

Dinoflagellate abundance was most influenced by water temperature and depth (Fig. 3d). It was also noted that, though water temperature was closely related, its intensity of influence was lesser (Table 3) than the depth, salinity, total alkalinity, and transparency (Table 3 and Fig. 3d). A strong negative relation with silicate concentration was also indicated (PC1 in Table 3 and Fig. 3d), signifying the dependence of dinoflagellates on diatoms as food (Jacobson and Anderson 1986). The northern sector has less representation of dinoflagellates, except during monsoon season forming a single cluster (Fig. 5d). This was due to the low abundance of dinoflagellate species, specifically like *Dinophysis caudata*, *Lingulodinium polyedrum*, *Prorocentrum minimum*, etc. Thus, the CCA (Fig. 5d, Table 4) does not include the monsoon season for northern sector. Figure 5d shows three major clusters of samples, one related to total alkalinity, another cluster highly influenced by silicate concentration, depth, and water temperature and the last cluster influenced by salinity. Dinoflagellate distribution is known to be influenced by environmental parameters like light, temperature, and salinity (Alkawri and Ramaiah 2010; D’Costa et al. 2008). The average temperature of the lagoon did not vary widely during the study period, while the salinity across the sectors and over seasons showed considerable variations, suggesting salinity might be a major influence on distribution of dinoflagellate species in the lagoon. Salinity dependent distribution of dinoflagellate species of the lagoon have been discussed by Mukherjee et al. (2016) suggesting salinity regime as important to maintain the dinoflagellate species diversity and abundance in the lagoon. Species of genus *Alexandrium* were most influenced with depth, silicate concentration, and water temperature, whereas spatial distribution of the remaining species was most influenced by salinity regime of the lagoon, preferring sites with higher salinity. Most of the species of *Neoceatium*, *Lingulodinium*, *Gonyaulax*, etc. were prevalent and abundant in outer channel during premonsoon period. *Dinophysis caudata*, *Noctiluca scintillans*, and *Protoperidinium* spp. were most influenced by total alkalinity. Dinoflagellates exhibited a strong negative relation with silicates (Fig. 3d). Species like *Prorocentrum belizeanum*, *P. minimum*, *Alexandrium ostenfeldii*, etc. in Fig. 5d are less affected by silicate concentration, an observation also supported by Gong and Hu (2014). Their study specifically showed that dominance of *Alexandrium* sp. and *Prorocentrum minimum* was less affected by silicate concentration, an observation (Fig. 3d) also incurred from the present work. Species like *Dinophysis caudata, Noctiluca scintillans*, *and Protoperidinium* spp. are negatively related to the silicate concentration. Silicate is mostly utilized by bacillariophytes for their structural and functional purposes, producing shells that are hard to ingest for dinoflagellates (Zhang et al. 2017). *Noctiluca scintillans* were examined to prefer consuming low silicate diatoms (Zhang et al. 2017) and thus negatively relate to silicate (Fig. 3d). Thus, the negative relation with silicate (Fig. 3d) is an indication that abundance of dinoflagellates is generally divergent to bacillariophytes on choosing low silicate diatoms to feed upon.

### Rotifera

Salinity and total alkalinity negatively influenced rotifer abundance in the lagoon (Table 3 and Fig. 3e) along with positive effect of silicate and phosphate. A negative relationship of rotifer abundance with total alkalinity and transparency (PC1 and PC 2 of Table 3) also seems to be determining factor rotifer abundance in the lagoon. The species level interaction of rotifer (Fig. 5d) showed salinity, total alkalinity, and Free CO_2_ to be the most important parameters influencing species distribution in the lagoon. Figure 3e revealed a very strong but negative correlation of rotifer abundance with total alkalinity, specifically in outer channel during postmonsoon period. The CCA analysis indicated rotifers to be temporally more distinct with two clusters (Fig. 5e), one of monsoon influenced by free CO_2_ and the other of postmonsoon influenced by total alkalinity. Salinity and nitrate during premonsoon, transparency, nitrate and salinity during postmonsoon period, and total alkalinity and salinity during monsoon were most deterministic parameters. *Lecanestyrax* was highly dependent on salinity whereas few species like *Lecane batilifer*, *Brachionus bidentata*, *Monostyla bulla* and *Monostyla luna* were found to have more affinity to nitrate and transparency. Other species like *Hexarthra* sp., *Lecaneinopinata*, *Filinia* sp. *Brachionusfalcatus* etc. were more dependent on fluctuation of free CO_2_. Rotifer population had strongest negative correlation with nitrate (Fig. 3e and PC2 of Table 3). This negative relation could be a possibility of rotifers dependence on nitrate-influenced microphytoplankton like cyanophyceae. Thus, nitrate can be a limiting factor for rotifer population too. Various species of *Brachionus* have shown both negative and positive (*Lecanebatillifer*, *Brachionus bidentata*, *Monostyla bulla*, *Monostyla luna*) influence of nitrate on them (Fig. 5e). The species like *Monostyla luna* and *Monostyla bulla* that depend on nitrate (Fig. 5e) are known specifically to depend on blue green algae (Green 2003) that in turn are nitrate dependent. Of all the sectors, the outer channel showed least abundance of rotifers with presence of only Brachionidae and Lecanidae. Saline or brackish water species like *Brachionus plicatilis* and *Brachionus falcatus* (Epp and Winston 1977) recorded from outer channel and southern sector during postmonsoon period were possible entry from the Bay of Bengal, through the sea-mouth of the lagoon, as they are present in Bay of Bengal (Mohapatra and Patra 2012).

### Tintinninae

Tintinnids have been noted as one of the most neglected groups of microplankton in the lagoon with regard to its diversity, abundance or ecological importance (Mukherjee et al. 2015). Tintinnid abundance was influenced by various environmental parameters; salinity followed by water temperature and depth were the most important among these (Fig. 3f). With only 39.52% of total variation explained (Table 2), the loadings of PC1 and PC2 (Table 3) showed highest negative influence of water temperature followed by salinity and depth (Fig. 3f). Water temperature has also been regarded as one of the most important abiotic influences on tintinnids by Dolan and Pierce (2013), but in tropical localities with less significant fluctuations in water temperature, it is not expected to have measurable effect. Moreover salinity regime of the lagoon increases during premonsoon period (Table 1) and has a positive effect on abundance of these species (Figs. 2e and 3). In tandem, water temperature too should have had a positive effect; instead most species, except few *Tintinnopsis* sp., have shown negative influence of increasing water temperature suggesting minor changes of water temperature can affect distribution of this group of protozoans. A strong relation with silicate concentration, pH, and dissolved oxygen was also observed. Figure 5f showed three major clusters, one with most species of tintinnids, viz. *Tintinnopsis gracilis*, *Stenosemella* sp., *Favella campanula*, *Eutintinnus* sp., and others influenced by salinity. Most species of *Tintinnopsis* viz., *T. radix*, *T. fimbriata*, *T. spiralis*, *T. directa*, etc. and *Favella adriatica*, belonging to the second cluster were related to water temperature and silicate (Fig. 5f). The third cluster formed of other species of tintinnopsis viz. *T. tocantinensis*, *T. cylindrica*, *T. parvula*, etc. were more influenced by pH and DO. As also indicated from Fig. 4f, tintinnid growth rate are inversely related to pH (Taniguchi and Kawakami 1983), but, Fig. 5f, indicated a few specific species like *Tintinnopsis karjakinensis* and *T. cylindrica* to have strong positive relation with pH and DO in the lagoon. As emphasized by Biyu (2000) tintinnid abundance and biomass remains lower but diversity higher in macrophyte rich areas of the lagoon. This attributed to the fact that *Tintinnopsis* spp. were regularly recorded in good numbers from central sector of the lagoon (Fig. 5f) which has abundant macrophytes (Mohanty et al. 2009; Srichandan et al. 2015) causing increases in pH and DO (Table 1). Though tintinnids are marine species, their distribution in the lagoon was not only determined by salinity but other important water parameters too. Nevertheless, genus *Tintinnopsis* that formed the highest species diversity in the lagoon was more influenced by water temperature, pH, and DO rather than salinity. As reported by Burns (1983), all *Tintinnopsis* sp. use silica grains to build their lorica. Species of *Tintinnopsis* that are more agglomerated with silica or diatoms shells on lorica reflected strong relation to silicate (Fig. 5). An another important observation was the distribution of three species of *Favella*, wherein *Favella campanula* showed influence of salinity, *F. adriatica* was influenced by water temperature and silicate, but the distribution of *F. eherenbergii*, the most abundant species of the three did not show an influence of any of the water parameters and thus remained unexplained.

With such large plankton diversity in the lagoon and as the results reports each group of plankton differently influenced by environmental parameters; analyzing total plankton dynamics to draw out crucial environmental inferences would be inappropriate. Each group of microplankton studied being very distinct in their seasonality, their dynamics studied with respect to environmental parameters unveiled the biotic-abiotic relationship of the ecosystem at primary level. The coccus cyanophytes have a nitrate reducing property and are known for recycling nutrients in waste water. Appropriate abundance of these species can keep the lagoon free from often faced excess nutrient loads (Panigrahi et al. 2007). Although *Noctiluca scintillans* is present in the lagoon and its harmful algal blooms (HAB) have been reported from the east coast (Baliarsingh et al. 2016) connected to the lagoon. As discussed earlier, the species dynamics is limited by lesser abundance of low silicate diatoms and less fluctuating total alkalinity (Fig. 5d), providentially indicating why no HAB’s have occurred so far and yet possible in the lagoon. Thus, studies on species-specific dynamics based on environmental parameters would be essential for assessing the lagoon ecosystem status. The inter-specific relations revealed through the study can help determine environmental parameters most necessary for maintaining of ecological balance. For instance, the silica polymerization inside the silica deposition vesicle of diatoms is dependent on salinity and pH (Vrieling et al. 1999) and thus are important parameter for not only sustaining the diversity (of diatom and tintinnids) but also preventing ecological disturbances regulating HAB’s of potential cyanophytes and dinoflagellates; as discussed earlier are dependent on bacillariophytes abundance (Fig. 3d).The diversity and abundance of rotifers that are very crucial to feeding of fish larvae (Lubzens et al. 1989) were limited in outer channel (Fig. 2) due to high salinity (Table 1). Thus, abundance and dynamics of specific species rotifers like *Brachionus plicatilis* and *Brachionus falcatus* play a vital role in outer channel. Considered as one of the most important spawning and nursing grounds for fishes (Jhingran 1963), continous siltation and disconnection to sea (Ghosh and Pattnaik 2005) can cause changes in dynamics of important plankton groups or species and in turn fish recruitment of outer channel. The study presses on the fact that parameters like pH and DO reported as less important (Srichandan et al. 2015) looked spatiotemporally most stable (Table 1) and obscure (Fig. 3a–f) on plankton dynamics of Chilika. But species-specific effect of individual microplankton groups like cyanophyceae and tintinninae (Fig. 5b, f) show pH and DO as prominent influencing parameter of the lagoon. With detailed bio-statistical analysis, it can be determined that the most deterministic physicochemical parameters influencing the dynamics of microplankton in the lagoon were salinity, pH, and DO. Of the nutrient parameters, nitrate and silicate were the limiting factors for growth and sustenance of microplankton (Fig. 5). Thus the study concluded that the abundance and distribution of microplankton can vary to individual genre and species level. Spatio-temporal variations with respect to the changes in the environmental parameters of a dynamic ecosystem like Chilika lagoon would be better understood when studied as individual groups rather than as total plankton abundance.
